# Draft Genome Sequences of *Mycobacterium kansasii* Strains 1010001454, 1010001458, 1010001468, 1010001493, 1010001495, and 1010001469, Isolated from Environmental Sources

**DOI:** 10.1128/genomeA.00456-16

**Published:** 2016-06-02

**Authors:** Dominik Strapagiel, Paulina Borówka, Błażej Marciniak, Zofia Bakuła, Jakko van Ingen, Aleksandra Safianowska, Anna Brzostek, Jarosław Dziadek, Tomasz Jagielski

**Affiliations:** a Biobank Lab, Department of Molecular Biophysics, University of Łódź, Łódź, Poland; b Department of Anthropology, University of Łódź, Łódź, Poland; c Department of Applied Microbiology, Institute of Microbiology, Faculty of Biology, University of Warsaw, Warsaw, Poland; d Department of Medical Microbiology, Radboud University Medical Center, Nijmegen, The Netherlands; e Department of Internal Medicine, Pulmonology and Allergology, Warsaw Medical University, Warsaw, Poland; f *Mycobacterium* Genetics and Physiology Unit, Institute of Medical Biology, Polish Academy of Sciences, Łódź, Poland

## Abstract

*Mycobacterium kansasii* belongs to the nontuberculous mycobacteria (NTM) and causes opportunistic infections with both pulmonary and extrapulmonary manifestations. Here, we report the draft genome sequences of six environmental *M. kansasii* strains, designated 1010001495 (type I), 1010001469 (type II), 1010001468 (type III), 1010001458 (type IV), 1010001454 (type V), and 1010001493 (type V), originally isolated in five different European countries.

## GENOME ANNOUNCEMENT

*Mycobacterium kansasii* belongs to the nontuberculous mycobacteria (NTM) and causes opportunistic infections with both pulmonary and extrapulmonary manifestations. Similar to other NTM, *M. kansasii* infections are thought to be acquired from the environment rather than a result of human-to-human transmission. Although the natural reservoir of *M. kansasii* has not definitively been identified, the pathogen has frequently been recovered from municipal tap water, which is considered to be its major environmental source (1, 2).

Globally, *M. kansasii* is the sixth most commonly isolated NTM species. In Poland, Slovakia, and the United Kingdom, it even ranks first, with a frequency of isolation of 35%, 36%, and 11%, respectively, compared to a mean isolation rate of 5% in Europe (2). Given the environmental source of transmission, the culture of *M. kansasii* from clinical samples from nonsterile sites may not represent true infection but transient colonization or inadvertent contamination.

Seven (I to VII) subtypes exist within the *M. kansasii* species. Of these, types I and II are most prevalent and have been associated with human disease, whereas types III to VII are predominantly environmental saprophytes (3 – 5). The genetic background of either the pathogenic or commensal/neutral phenotype of different *M. kansasii* types has not yet been explored. While the complete genome sequence of the *M. kansasii* type I strain ATCC 12478 was released in 2015, no sequences of environmental *M. kansasii* types have been released.

Here, we announce the draft genome sequences of six environmental *M. kansasii* strains, designated 1010001495 (type I), 1010001469 (type II), 1010001468 (type III), 1010001458 (type IV), 1010001454 (type V), and 1010001493 (type V), originally isolated from either water or soil samples from five different European countries.

Genomic DNA was extracted and purified using the protocol of van Embden et al. (6). Illumina paired-end libraries were prepared with 1 ng of genomic DNA using the Nextera XT kit. Whole-genome shotgun sequencing was performed on the NextSeq 500 platform at a read length of 2 × 150 bp. A total of 7095852, 9152184, 6836762, 7565072, 7618714, 8905318 reads and coverages of 25×, 37×, 24×, 29×, 28×, and 34× for strains 1010001454, 1010001458, 1010001468, 1010001493, 1010001495, and 1010001469, respectively, were generated. The draft genomes were *de novo* assembled by SPAdes 3.7.1 (7), with manual editing using FA_TOOL (8). The assemblies for strains 1010001454, 1010001458, 1010001468, 1010001493, 1010001495, and 1010001469 included 345, 187, 164, 173, 140, and 162 contigs >1,000 bp, respectively. Annotation was carried out using the NCBI PGAP (http://www.ncbi.nlm.nih.gov/genome/annotation_prok/). The assemblies for strains 1010001454, 1010001458, 1010001468, 1010001493, 1010001495, and 1010001469 contained 5,165, 5,439, 5,186, 5,064, 5,265, and 5,264 predicted protein-coding sequences, respectively. The total lengths of the contigs for each genome were 6,171,688 bp, 6,027,332 bp, 6,141,835 bp, 5,627,124 bp, 6,358,240 bp, and 6,266,032 bp, respectively, with a G+C content of 66.11 to 66.38%.

### Nucleotide sequence accession numbers.

The draft genome sequences of the *M. kansasii* strains 1010001454, 1010001458, 1010001468, 1010001493, 1010001495, and 1010001469 were deposited at DDBJ/EMBL/GenBank under the accession numbers LWCH00000000, LWCI00000000, LWCJ00000000, LWCK00000000, LWCL00000000, and LWCM00000000, respectively. The versions described in this paper are the first versions, LWCH01000000, LWCI01000000, LWCJ01000000, LWCK01000000, LWCL01000000, and LWCM01000000.
